# Assesment of the QuickSee wavefront autorefractor for characterizing refractive errors in school-age children

**DOI:** 10.1371/journal.pone.0240933

**Published:** 2020-10-28

**Authors:** Andrea Gil, Carlos S. Hernández, Pablo Pérez-Merino, Marcos Rubio, Gonzalo Velarde, María Abellanas-Lodares, Ángeles Román-Daza, Nicolás Alejandre, Ignacio Jiménez-Alfaro, Ignacio Casares, Shivang R. Dave, Daryl Lim, Eduardo Lage

**Affiliations:** 1 Department of Electronics and Communications Technology, Universidad Autónoma de Madrid, Madrid, Spain; 2 Instituto de Investigación Sanitaria Fundación Jiménez Díaz, Madrid, Spain; 3 PlenOptika, Inc., Boston, MA, United States of America; 4 Hospital Ruber Juan Bravo, Madrid, Spain; 5 Department of Ophthalmology, Hospital Universitario Fundación Jiménez Díaz, Madrid, Spain; University of Missouri-Columbia, UNITED STATES

## Abstract

**Purpose:**

To assess the performance of an open-view binocular handheld aberrometer (QuickSee) for diagnosing refractive errors in children.

**Methods:**

123 school-age children (9.9 ± 3.3 years) with moderate refractive error underwent autorefraction (AR) with a standard desktop device and subjective refraction (SR), with or without cycloplegia to determine their eyeglass prescription. Measurements with QuickSee (QS) were taken in 62 of these patients without cycloplegia (NC), and in 61 under cycloplegia (C). Differences in refraction values (AR vs SR vs QS) as well as the visual acuity (VA) achieved by the patients with each method (QS vs SR) were used to evaluate the performance of the device in measuring refractive error.

**Results:**

The spherical equivalent refraction obtained by QS agreed within 0.5 D of the SR in 71% (NC) and 70% (C) of the cases. Agreement between the desktop autorefractor and SR for the same threshold was of 61% (NC) and 77% (C). VA resulting from QS refractions was equal to or better than that achieved by SR procedure in 77% (NC) and 74% (C) of the patients. Average improvement in VA with the QS refractions was of 8.6 and 13.4 optotypes for the NC and C groups respectively, while the SR procedure provided average improvements of 8.9 (NC) and 14.8 (C) optotypes.

**Conclusions:**

The high level of agreement between QuickSee and subjective refraction together with the VA improvement achieved in both study groups using QuickSee refractions suggest that the device is a useful autorefraction tool for school-age children.

## Introduction

Tackling visual impairment in childhood is a major global concern because it affects a child’s learning process, self-perception, life skills and overall development [1]. The World Health Organization reported in 2018 that nearly 1.3 billion people worldwide had vision impairment, the bulk of which are located in low- and middle-income countries [2]. According to these sources, around 12.8 million children below the age of 15 years old [3] were visually impaired due to uncorrected refractive errors (UREs) [4], which remains the leading cause of visual disability among school-age children [4–6].

Although prescription eyeglasses are a cost-effective remedy for UREs, the reality is that many children do not receive appropriate, or even basic eye care. Consequently, they are not able to benefit from refractive correction. In low-resource settings and areas of conflict, the main causes of UREs have been related to a shortage of eye care professionals, and to a lack of access to resources for vision screening [4, 7, 8]. Even in developed countries with greater access to eyecare, significant prevalence of UREs among children have been found (e.g. 13.1% in the school district of Philadelphia [9], or 7.2% in Ireland [10]), and is expected to increase in the coming years [11]. This may indicate that the problem is not only related to a lack of resources, but also to eye care awareness.

QuickSee (PlenOptika Inc, USA) is a handheld wavefront autorefractor which has recently become commercially available and may be of particular interest for pediatric use due to several features of note. First, the device is open-view–the user looks through the device at a distant target during the measurement–a feature that has effectively been used to reduce accommodation during autorefraction [12–15]. Second, this autorefractor measures both eyes simultaneously, which is of particular importance for child vision screening as it allows for greater relaxation of accommodation, while better replicating normal viewing conditions [16]. This unique combination of open-view and binocular measurement avoids any differences in patient accommodation that may arise between sequential readings of each eye, thus eliminating any type of measurement-induced anisometropia. Third, QuickSee measurements are taken dynamically to track eye position and accommodative state [17]. This feature makes it possible to issue warnings when anomalies such as excessive accommodation or misalignments are detected. Finally, measurements with QuickSee can be acquired without any restrictions on ambient lighting conditions. This is a significant advantage over other portable refraction devices (e.g. photoscreeners), which can typically only be used under dark ambient or controlled lighting conditions.

Recent clinical studies of QuickSee have focused on comparing the agreement of the device with SR in several adult populations. While it was found that QuickSee provided excellent agreement with SR in those groups [14, 15, 17, 18], the performance of the device in children, a significantly more challenging population, remained unknown. This work presents the first evaluation of the performance of this binocular wavefront autorefractor in characterizing refractive errors in pediatric populations.

## Material and methods

### Study population

All study participants were recruited from the standard pediatric population visiting the Ophthalmology Department of Fundación Jiménez Díaz Hospital (Madrid, Spain). Inclusion criteria were: (1) ages between 4 and 16 years old, (2) best-corrected visual acuity (VA) of 0.3 LogMAR units (20/40) or better in each eye, and (3) a refractive error within the measurement range of QuickSee (-10 D to +10 D of Sphere and -6 D to +6 D of astigmatism). Exclusion criteria were: (1) use of systemic or ocular drugs that may affect vision, and (2) history of surgery or eye disease other than strabismus. The study was integrated into the clinical workflow of the hospital and written consent from a parent or legal guardian was mandatory. The research was approved by the institutional review board of the Hospital and followed the tenets of the Declaration of Helsinki.

### Equipment kit

The table-mounted Topcon KR 8800 (Topcon Corporation, Tokyo, Japan) and the portable QuickSee autorefractors were used for objectively assessing refractive errors. The Topcon KR 8800 is a closed-view, benchtop device (20 kg), based on rotary prism technology that only acquires monocular measurements (one eye at a time). To mitigate accommodation and instrument induced myopia during measurements, the device uses a proprietary auto-fogging function. QuickSee is an open-view wavefront aberrometry-based handheld autorefractor (1.2 Kg) that can acquire measurements binocularly (both eyes simultaneously), and monocularly. Monocular measurements can be taken when difficulties are faced in achieving proper simultaneous alignment of both eyes, such as being caused by facial asymmetries, or the presence of cataracts or strabismus. Even in monocular measurement mode, the patient looks through the device with both eyes open during the data acquisition.

Visual acuity measurements were taken under constant lighting conditions with an Aurochart digital vision chart (Aurolab, Tamil Nadu, India) configured to show the ETDRS (Early treatment Diabetic Retinopathy study) [19] LogMAR chart adjusted for a 4-m testing distance. A tumbling E illiterate LogMAR chart was projected in the digital vision chart for younger participants.

### Experimental protocol

All patients included in the study underwent standard pediatric clinical refraction consisting of autorefraction and subjective refraction to determine their eyeglass prescription. The first cohort of patients were measured with the KR 8800 autorefractor and with QuickSee under non-cycloplegic (NC) conditions. After autorefraction, an experienced pediatric optometrist performed subjective refraction with trial lenses using the KR 8800 reading as starting point. A second cohort of patients underwent the same study protocol after instillation of a cycloplegic agent, Cyclopentolate 1% (C).

In both study groups, the main outcome measures were the power vector components of the refraction (M, J_0_ and J_45_) measured by the KR 8800, QuickSee, and by subjective refraction. Data collection also included three monocular/binocular visual acuity measurements (without any correction, and with trial frames set to the SR and QuickSee refractions), which were recorded using the LogMAR scale.

### Data analysis

Only data from the right eye is reported in the analysis due to the correlation of refractive errors in both eyes. Agreement among refraction methods was assessed comparing QuickSee measurements with the results obtained by the KR 8800 autorefractor and SR (with or without cycloplegia for the C and NC groups, respectively). Autorefraction accuracy was also evaluated by determining the number of eyes in which agreement between SR and each autorefractor was within 0.5 D and 1 D thresholds for M and within 0.25 D and 0.5 D for J_0_ and J_45_. A Bland-Altman analysis (mean difference, standard deviation, and 95% confidence intervals) of power vector components (M, J_0_, and J_45_) was also performed to evaluate the agreement between both autorefractors (QuickSee vs KR 8800), and between the autorefractors and SR (QuickSee vs SR, KR 8800 vs SR). Intraclass Correlation Coefficients (ICC) were used in all cases as an additional figure of merit to assess the similarity between both autorefractors and SR in each group.

Monocular VA measurements were used to perform an analysis comparing VA after corrections based on subjective and QuickSee refractions. The VA analysis also includes a comparison between refraction methods to determine the number of patients achieving VAs of 20/25, 20/20 or better, and the average improvement in VA achieved by SR or QuickSee refractions with respect to uncorrected VA.

## Results

### Characteristics of study population

A total of 136 patients participated in the study (71 noncycloplegic and 65 cycloplegic). There was incomplete information from 10 of those patients (6 NC and 4 C) due to clinical refraction being performed by retinoscopy (3), or with other benchtop autorefractors (7). Apart from the previous 10 patients, it was not possible to obtain accurate readings in another 3 noncycloplegic patients with QuickSee. An accommodation detection warning was indicated by the device after 3 measurement attempts in those patients. This warning is given when refractive power fluctuates significantly during a measurement, such that the unaccommodated refractive status cannot be reliably determined. Thus, such measurements were considered unreliable and excluded from the study (as they would also be excluded in clinical use). None of the participants were untestable by QuickSee.

The average age of patients included in the statistical analysis was 9.74 ± 2.88 years for the NC group (n = 62), and 10.18 ± 3.17 years for the C group (n = 61). Refractive error classification of the population in Tables 1 and 2 is based on the subjective refraction results and is further broken down by age. Non-corrected sphere in the right eye of the NC cohort ranged from -4.25 to +6.75 D, and from -7.5 to +6.25 D in the C group. The cylindrical component of the refraction ranged from 0 to -4 D for the NC group and from 0 to -2.5 D in the C group.

**Table 1:** Refractive error in the right eyes of The NC group (N, %) categorized by age.

	Age Group			
	4–6 y/o	7–12 y/o	13–16 y/o	Total
**Hyperopia**	5 (8%)	23 (37%)	3 (5%)	31 (50%)
**0.5–3.5 D**	5 (8%)	22 (35%)	3 (5%)	30 (48%)
**>3.5 D**	0	1 (2%)	0	1 (2%)
**Myopia**	1 (2%)	11 (18%)	5 (8%)	17 (28%)
**0.5–1.5 D**	0	7 (11%)	1 (2%)	8 (13%)
**> 1.5 D**	1 (2%)	4 (7%)	4 (7%)	9 (15%)
**Astigmatism**	7 (11%)	28 (45%)	5 (8%)	40 (64%)
**0.5–1.5 D**	5 (8%)	21 (34%)	4 (6%)	30 (48%)
**> 1.5 D**	2 (3%)	7 (11%)	1 (2%)	10 (16%)
**Emmetropia**	0	3 (5%)	4 (7%)	7 (12%)

**Table 2:** Refractive error in the right eyes of The C group (N, %) categorized by age.

	Age Group			
	4–6 y/o	7–12 y/o	13–16 y/o	Total
**Hyperopia**	7 (11%)	21 (34%)	5 (8%)	33 (54%)
**0.5–3.5 D**	7 (11%)	18 (30%)	4 (7%)	29 (48%)
**>3.5 D**	0	3 (5%)	1 (2%)	4 (7%)
**Myopia**	0	10 (16%)	8 (13%)	18 (30%)
**.5–1.5 D**	0	6 (10%)	1 (2%)	7 (11%)
**> 1.5 D**	0	4 (7%)	7 (11%)	11 (18%)
**Astigmatism**	8 (13%)	22 (36%)	7 (11%)	37 (61%)
**0.5–1.5 D**	6 (10%)	19 (31%)	6 (10%)	31 (51%)
**> 1.5 D**	2 (3%)	3 (5%)	1 (2%)	6 (10%)
**Emmetropia**	1 (2%)	0	2 (3%)	3 (5%)

### Comparison of mean results in the study groups

Average spherical equivalent refractions for the NC and C groups are presented in Table 3, as well as the mean values of the cartesian and oblique components for each refraction method (Subjective, QS, AR). Mean spherical equivalent differences between non cycloplegic and cycloplegic QuickSee and subjective refraction are -0.29 D and 0.03 D, respectively. The same differences between the KR-8800 and SR are -0.35 D (NC) and 0.1 D (C). Further details, including intraclass correlation coefficients between SR and the two autorefractors are also shown in the table.

**Table 3:** Mean (±SD) refraction power for each refraction method under cycloplegic and noncycloplegic conditions.

		Spherical Equivalent (M)			Cartesian component (J_0_)			Oblique component (J_45_)		
	Study Group	Subjective	QuickSee	KR 8800	Subjective	QuickSee	KR 8800	Subjective	QuickSee	KR 8800
**Mean ± SD**	NC	-0.06 ± 1.73 D	-0.35 ± 1.52 D	-0.41 ± 1.94 D	0.36 ± 0.55 D	0.37 ± 0.47 D	0.33 ± 0.57 D	0.00 ± 0.17 D	0.00 ± 0.17 D	0.00 ± 0.20 D
	C	0.09 ± 2.25 D	0.06 ± 2.13 D	-0.01 ± 2.36 D	0.24 ± 0.36 D	0.34 ± 0.41 D	0.26 ± 0.37 D	0.01 ± 0.17 D	0.03 ± 0.24 D	0.02 ± 0.21 D
**ICC**	NC	-	0.93	0.97	-	0.96	0.98	-	0.86	0.94
	C	-	0.98	0.98	-	0.82	0.97	-	0.62	0.85

ICC, Intraclass correlation coefficients; SD, Standard deviation; NC, Noncycloplegic group; C, Cycloplegic group.

### Bland-Altman comparison

**Fig 1:**
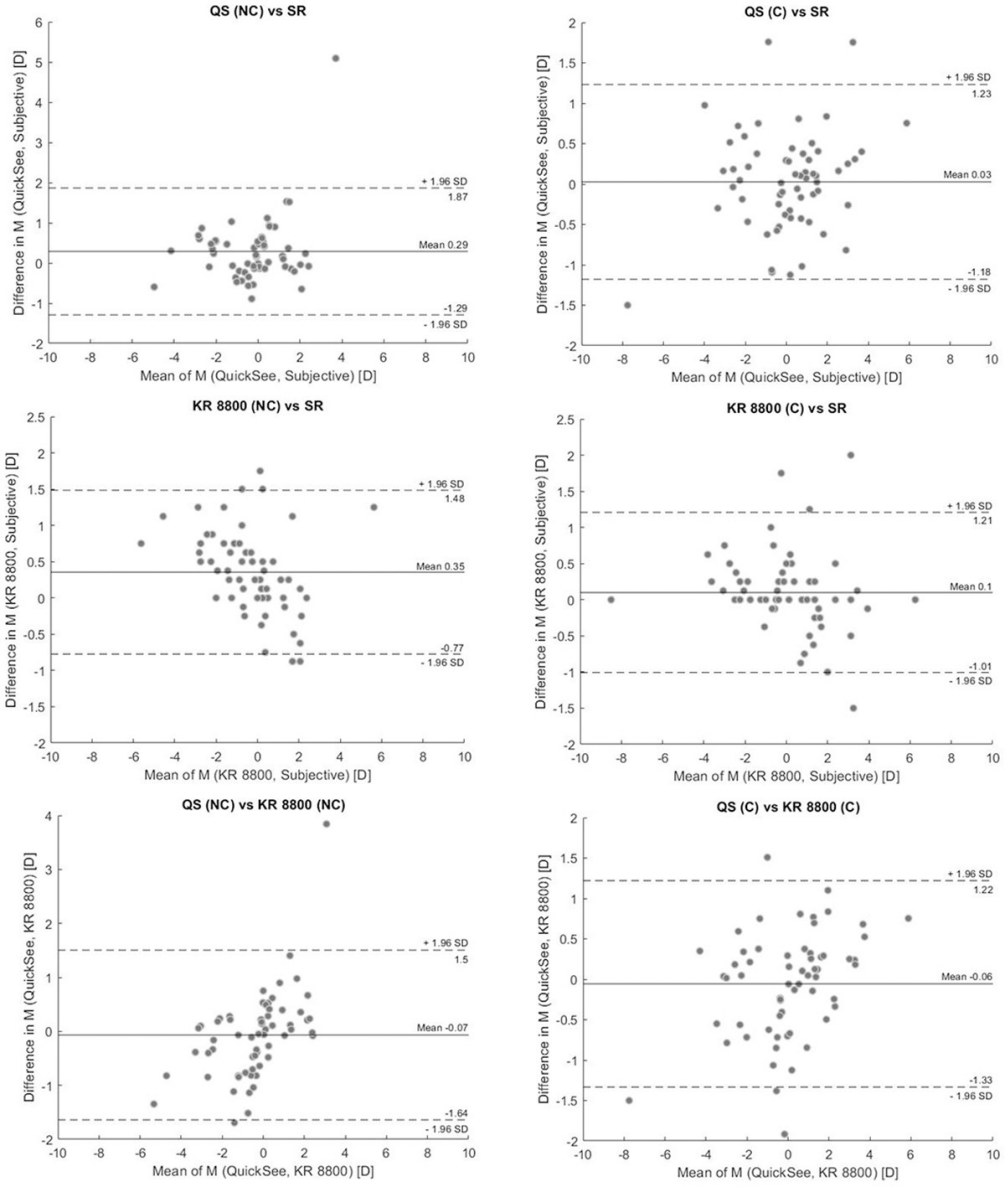
Bland-Altman plots showing the agreement among spherical equivalent refractions measured with the KR 8800, QuickSee and subjective refractions in cycloplegic (right) and noncycloplegic (left) conditions.

**Fig 2:**
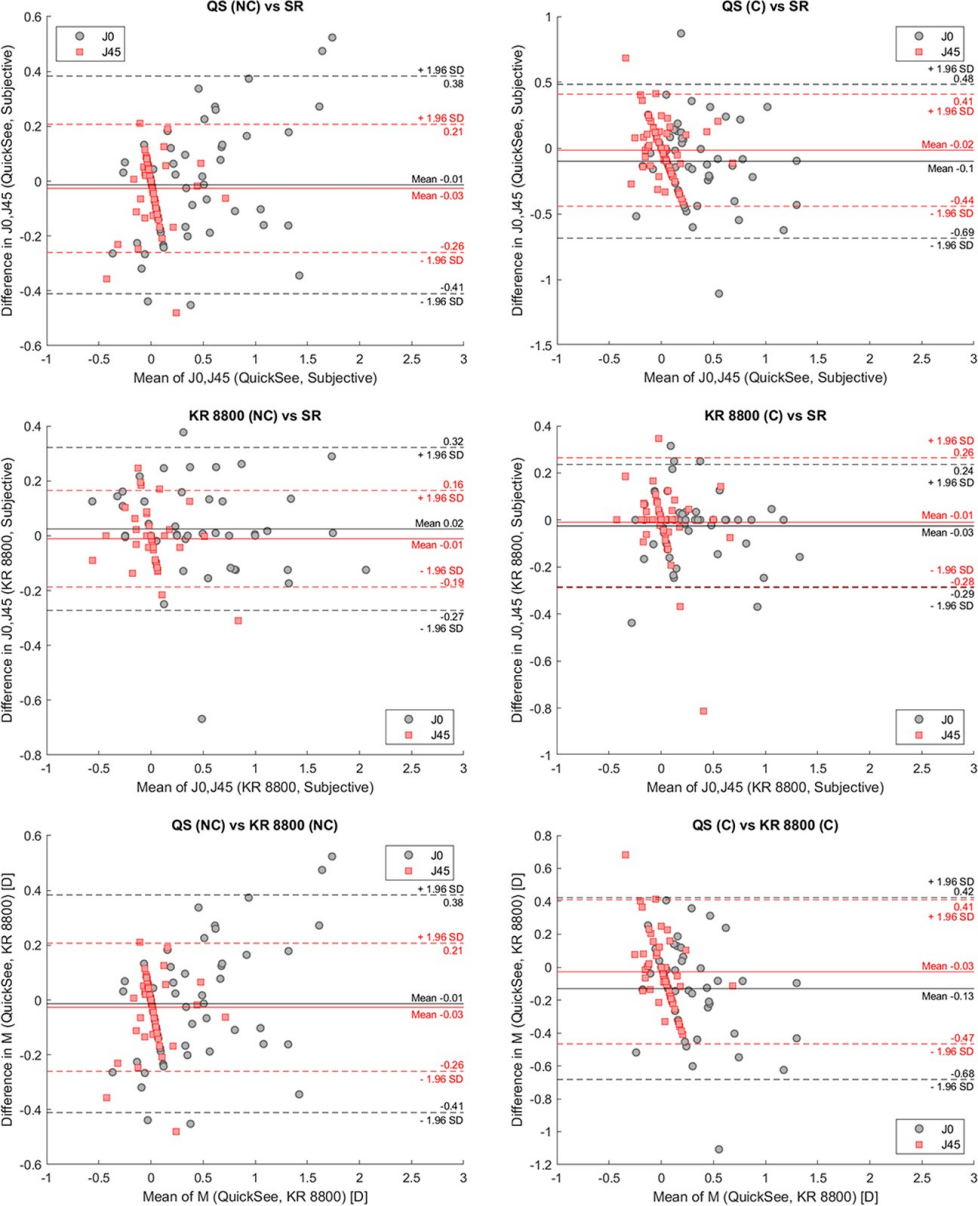
Bland-Altman plots showing the agreement among cylindrical refractions measured with the KR 8800, QuickSee and subjective refractions in cycloplegic (right) and noncycloplegic (left) conditions.

The Bland-Altman analyses of the data (Figs 1 and 2) indicated a bias and limits of agreement (LoA) between SR and QuickSee of 0.29 ± 1.58 (M), -0.01 ± 0.39 (J_0_), and -0.03 ± 0.24 D (J_45_) when the study protocol was performed without cycloplegia (NC group). In the case of cycloplegic patients, the biases ± limits of agreement between QS and SR were 0.03 ± 1.20, -0.10 ± 0.58, and -0.02 ± 0.43 D for M, J_0_ and J_45_, respectively. The noncycloplegic measurements of the KR 8800 autorefractor had a bias with SR of 0.35, 0.02, and -0.01 D with limits of agreement of ± 1.13, ± 0.30, and ± 0.17 D for M, J_0_, and J_45_, respectively. Differences between the KR 8800 and SR in the C group resulted in biases and limits of agreement of 0.08 ± 1.09 (M), -0.03 ± 0.26 (J_0_), and -0.01 ± 0.27 D (J_45_).

### Agreement with subjective refraction

QuickSee showed an agreement with SR within 0.5 D of 71% (M), 100% (J_0_) and 100% (J_45_) in the NC group. For the C group, an agreement of 70% (M), 92% (J_0_) and 98% (J_45_) was found when the same threshold (0.5 D) was applied. The percentage of eyes within 0.5 D for the KR 8800 autorefractor were 61% (M), 98% (J_0_) and 100% (J_45_) in the NC group, and 77% (M), 100% (J_0_) and 98% (J_45_) for the C group. With a higher difference threshold of 1 D, the number of eyes for M was 92% (NC) and 89% (C) for the QuickSee, and 85% (NC) and 90% (C) for the KR 8800. A complete report of the results obtained in this comparison is given in Table 4 for both study groups.

**Table 4:** Percentage agreement between autorefractors and subjective refraction.

	NC Group		C Group	
	QuickSee	KR 8800	QuickSee	KR 8800
**M (≤ 0.5 D / ≤ 1 D)**	71% / 94%	61% / 87%	70% / 90%	77% / 93%
**J**_**0**_ **(≤ 0.25 D / ≤ 0.5 D)**	85% / 100%	95% / 98%	70% / 92%	93% / 100%
**J**_**45**_ **(≤ 0.25 D / ≤ 0.5 D)**	97% / 100%	98% / 100%	79% / 98%	95% / 98%

NC, Noncycloplegic group; C, Cycloplegic group.

### Visual acuity analysis

**Fig 3:**
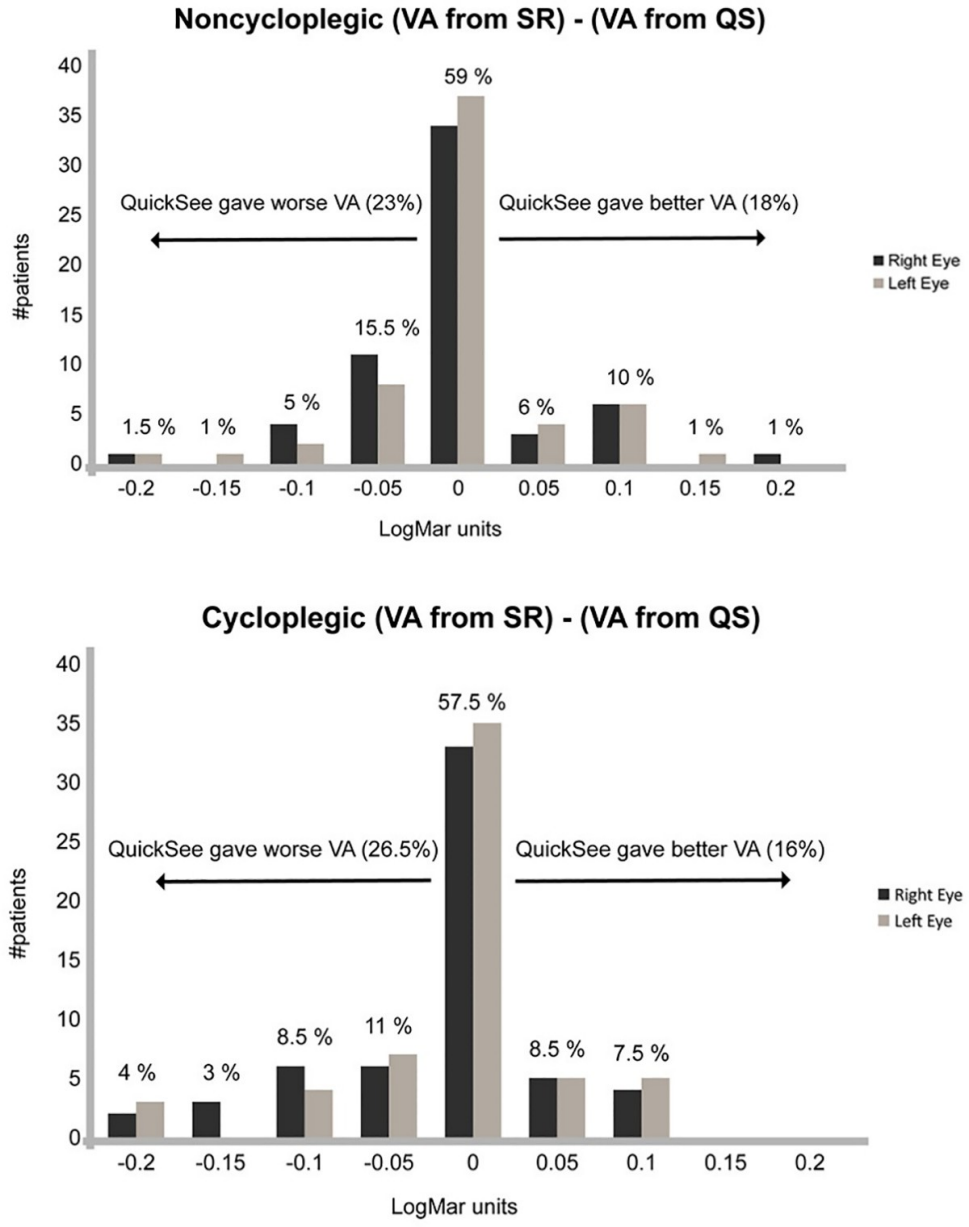
Proportion of noncycloplegic and cycloplegic patients in which the QuickSee refractions provided worse, better, or equal VA compared to SR.

The monocular average VA for the right eye (NC) was 0.20 ± 0.23, 0.02 ± 0.08, and 0.03 ± 0.08 LogMAR units for non-corrected, subjective and QuickSee refractions, respectively. This implies an average improvement of 0.179 LogMAR units (8.9 optotypes) for SR and 0.173 LogMAR units (8.6 optotypes) for QuickSee. For the cycloplegic group, average visual acuities were 0.33 ± 0.29 (non-corrected), 0.03 ± 0.05 (SR) and 0.06 ± 0.09 (QS) LogMAR units. Average improvement in visual acuity with trial lens set to the SR result was 0.297 LogMAR units (14.8 optotypes), while QuickSee itself provided average improvement of 0.268 LogMAR units (13.4 optotypes). Results for left eyes are not reported because of the high correlation with the right eyes results. In any case, Fig 3 contains detailed information for both eyes showing the proportion of cases in which VA provided by QuickSee is better, worse or the same as the subjective one. Overall, we found that corrected VAs based on QuickSee refraction were equal to or better than those achieved with the standard clinical procedure in 77% and 74% of the patients in the NC and C groups, respectively.

In the NC group, corrections based on QuickSee for the right eye provided 20/20 or better VA in 62% of cases and 20/25 or better VA in 93% of the patients. This is compared to 25% (20/20) and 48% (20/25) for uncorrected VA, and 80% (20/20) and 97% (20/25) achieved by SR. In the group measured under cycloplegic conditions 20/20 or better VA was achieved by 14%, 51%, and 37% of the patients for uncorrected, SR and QuickSee refractions, respectively, while 20/25 or better VAs were obtained in 30% (uncorrected), 95% (SR) and 88% (QuickSee) of the patients.

## Discussion

Despite the challenging subject population in this study (Tables 1 and 2), and the fact that the KR 8800 autorefractor was used as the starting point for the SR procedure, QuickSee demonstrated agreement with SR that is comparable to that achieved by the benchtop autorefractor.

In terms of spherical equivalent refraction (Fig 1, Table 4), both autorefractors showed a tendency towards minus over correction in the NC group, which was not found in the group tested under cycloplegia. In the NC group, the spherical equivalent refraction provided by QuickSee agreed within 0.5 D of SR in 71% of the patients, which is significantly better than the accuracy achieved by the KR 8800 in this group (61%). This finding suggest that the open-view design of QuickSee was more effective than the auto-fogging mechanism of the AR in mitigating the effect of accommodation, which is also in agreement with previous works comparing open and closed field-of-view autorefractors [20, 21]. In the cycloplegic group, the percentage of eyes with M within 0.5 D of SR for the KR 8800 increased significantly compared to the agreement achieved in the NC group (61% NC vs 77% C), while such differences between groups were not observed for QuickSee (71% NC vs 70% C). In terms of astigmatism, both autorefractors performed similarly well under non-cycloplegic conditions. However for the cycloplegic group, there was a reduction in the agreement between the astigmatic components (J_0_ and J_45_) measured by QuickSee, those measured by the KR 8800, and SR (Table 4 and Fig 1). These average differences, although noticeable (0.08 D and 0.01 D for J_0_ and J_45_, respectively), were smaller than those reported by other authors comparing autorefractors and wavefront aberrometers in young adults under cycloplegic conditions [22, 23].

Average differences in corrected VA based on each refraction method (QuickSee and SR) for the right eye were small (0.005 and 0.03 LogMAR units for the NC and C groups, respectively) with the SR procedure providing better performance in terms of resulting VA. In the non-cycloplegic group, 77% of subjects achieved the same (59%) or better (18%) VA with QuickSee correction than that achieved with the standard clinical protocol (Fig 3). This was largely replicated in the cycloplegic group, in which 74% of patients achieved the same (57.5%) or better (16%) VA than that provided by standard clinical protocol. Note that in the cycloplegic group, the visual acuities achieved with all refraction methods were in general smaller than those achieved in the NC group, which is expected due to increased peripheral and spherical aberrations caused from the pupil dilation.

It is worth noting that the main limitation of this study was the use of different patients for evaluating the performance of the device under cycloplegic and non-cycloplegic conditions. This limitation was accepted in order to minimize impact to the patient throughput of the hospital by reducing disruption to their standard clinical workflow. Another limitation is related to the low number of patients with high refractive error (|M| ≥ 3.5 D) in both study groups (Tables 1 and 2). This relative paucity of high refractive errors in the data preclude full characterization of the performance of the device through its complete measurement range. This is also reflected in the initial uncorrected visual acuity, which is relatively good in both groups (0.2 and 0.33 LogMAR units for the NC and C groups respectively) compared to the current definition of low vision (0.5 LogMAR, [24]). Consequently, the range of improvement in VA achieved by corrections is lower than what would be expected in patients with higher refractive error. Despite these and other study limitations (e.g. total number of subjects), the high-level of agreement between QuickSee and subjective refraction, as well as the resulting VA achieved by the patients in both study groups, suggest that the device would serve as a useful autorefraction tool for pediatric populations. Furthermore, the device’s robust screening metrics, along with its ease of use and handheld form factor, support its use as a pediatric vision screening tool.

## Supporting information

S1 File (XLSX)
