# Correction: Assesment of the QuickSee wavefront autorefractor for characterizing refractive errors in school-age children

**DOI:** 10.1371/journal.pone.0270449

**Published:** 2022-06-21

**Authors:** Andrea Gil, Carlos S. Hernández, Pablo Pérez-Merino, Marcos Rubio, Gonzalo Velarde, María Abellanas-Lodares, Ángeles Román-Daza, Nicolás Alejandre, Ignacio Jiménez-Alfaro, Ignacio Casares, Shivang R. Dave, Daryl Lim, Eduardo Lage

The word "Assessment" is misspelled in the article title. The correct title is: Assessment of the QuickSee wavefront autorefractor for characterizing refractive errors in school-age children. The correct citation is: Gil A, Hernández CS, Pérez-Merino P, Rubio M, Velarde G, Abellanas-Lodares M, et al. (2020) Assessment of the QuickSee wavefront autorefractor for characterizing refractive errors in school-age children. PLoS ONE 15(10): e0240933. https://doi.org/10.1371/journal.pone.0240933
